# Deaths Associated with Hurricane Sandy — October–November 2012

**Published:** 2013-05-24

**Authors:** Mary Casey-Lockyer, Rebecca J. Heick, Caitlin E. Mertzlufft, Ellen E. Yard, Amy F. Wolkin, Rebecca S. Noe, Michelle Murti

**Affiliations:** American Red Cross; Div of Toxicology and Human Health Sciences, Agency for Toxic Substances and Disease Registry; Div of Environmental Hazards and Health Effects, National Center for Environmental Health; EIS Officer, CDC

On October 29, 2012, Hurricane Sandy* hit the northeastern U.S. coastline. Sandy’s tropical storm winds stretched over 900 miles (1,440 km), causing storm surges and destruction over a larger area than that affected by hurricanes with more intensity but narrower paths. Based on storm surge predictions, mandatory evacuations were ordered on October 28, including for New York City’s Evacuation Zone A, the coastal zone at risk for flooding from any hurricane (1). By October 31, the region had 6–12 inches (15–30 cm) of precipitation, 7–8 million customers without power, approximately 20,000 persons in shelters, and news reports of numerous fatalities (Robert Neurath, CDC, personal communication, 2013). To characterize deaths related to Sandy, CDC analyzed data on 117 hurricane-related deaths captured by American Red Cross (Red Cross) mortality tracking during October 28–November 30, 2012. This report describes the results of that analysis, which found drowning was the most common cause of death related to Sandy, and 45% of drowning deaths occurred in flooded homes in Evacuation Zone A. Drowning is a leading cause of hurricane death but is preventable with advance warning systems and evacuation plans. Emergency plans should ensure that persons receive and comprehend evacuation messages and have the necessary resources to comply with them.

Red Cross tracks deaths during disasters to provide services to surviving family members, including crisis counseling, assistance with disaster-related expenses, locating emergency housing, identifying recovery resources, and addressing disaster-related health needs. Red Cross volunteers search for reports of disaster-related deaths from sources such as funeral home directors, the Federal Emergency Management Agency (FEMA), hospitals, and news reports. Volunteers then obtain information about these deaths from sources including the medical examiner/coroner, physician, fire department/police, and family of the decedent (2).

Deaths included in this analysis were any Sandy-related death recorded on a Red Cross mortality form with a date of death up to November 30, 2012. Mortality forms included the decedent’s age, sex, race (white, black, Asian, other, or unknown), and date and location of death. Disaster-related deaths were categorized as direct or indirect. Directly related deaths are deaths caused by the environmental force of the disaster (e.g., wind or flood) or by the direct consequences of these forces (e.g., structural collapse). Indirectly related deaths are defined as deaths occurring in a situation in which the disaster led to unsafe conditions (e.g., hazardous roads) or caused a loss or disruption of usual services that contributed to the death (e.g., loss of electrical services) (2). Deaths without direct or indirect classification were reported as unknown or possibly related deaths. Daily counts of direct, indirect, and unknown/possibly related deaths were calculated based on the dates of each death. The characteristics of drowning deaths were compared with all deaths using chi-square tests of trend and t-tests. Home addresses of decedents whose drowning death occurred in the home were examined with respect to FEMA’s hurricane storm surge area (field-verified as of November 11, 2012 [3]) and known, geographically defined areas under evacuation order (i.e., New York City’s Evacuation Zone A) (1).

What is already known on this topic?

Despite advances in hurricane warning and evacuation systems, drowning remains one of the leading causes of hurricane-related deaths.

What is added by this report?

A total of 117 deaths related to Hurricane Sandy were reported via the American Red Cross mortality tracking system. Drowning was the leading cause, accounting for approximately one third of the deaths. More than half (52.5%) of the drowning deaths occurred in the decedent’s home; the majority of these homes were located in New York City’s Evacuation Zone A.

What are the implications for public health practice?

Drowning is a preventable cause of hurricane-related death. Hurricane response plans should ensure that persons receive and comprehend evacuation messages and have the necessary resources to comply with them.

A total of 117 deaths were reported on Red Cross mortality forms. The source of information for the mortality forms was a medical examiner/coroner for 94 (80.3%) cases and the family of the decedent for 10 (8.5%) cases (Table). Most deaths occurred in New York (53 [45.3%]) and New Jersey (34 [29.1%]); the other deaths occurred in Pennsylvania, West Virginia, Connecticut, and Maryland. The deaths occurred during October 28–November 29, 2012 (Figure 1). Approximately half of the deaths (60 [51.3%]) occurred on the first 2 days of the storm’s landfall, with a peak of 37 deaths on October 30, 2012.

Decedents ranged in age from 1 to 94 years (mean: 60 years, median: 65 years); 60.7% were male, and 53.8% were white. Of the 117 deaths, 67 (57.3%) were classified as directly related deaths, and 38 (32.5%) were indirectly related to the storm. Of the directly related deaths, the most common mechanism was drowning (40 [59.7%]), followed by trauma from being crushed, cut, or struck (19 [28.4%]). Poisoning was the most common indirectly related cause of death; of the 10 poisonings, nine were caused by carbon monoxide. Most directly related deaths occurred during the first few days of the storm, whereas indirectly related deaths continued from the day before the storm into the middle of November.

Comparing the 40 drowning deaths to all Sandy-related deaths, the age, sex, and race distributions of decedents were similar (Table). The majority of drowning deaths (29 [72.5%]) also occurred in the initial phase of the storm, during October 29–31. Twenty-one (52.5%) drowning deaths occurred in the decedent’s home, and 11 (27.5%) occurred outside; one person drowned in a flooded commercial building lobby, and another person drowned while intentionally swimming off a storm-affected beach. For six deaths, circumstances of the drowning were not available. The location of drowning deaths by state was significantly different (p<0.05) compared with all Sandy-related deaths. The majority of drowning deaths (32 [80.0%]) occurred in New York, whereas deaths in New York accounted for only 27.3% of nondrowning deaths. Twenty decedents drowned in flooded homes in New York, and home addresses for 18 (90.0%) of them were located in Evacuation Zone A (Figure 2); the other two decedents’ homes were in or near areas of flooding and near Evacuation Zone A. Notes written by Red Cross volunteers on these 20 deaths captured decedents’ reasons for not evacuating, such as “afraid of looters,” “thought Hurricane Irene was mild,” and “unable to leave because did not have transportation.”

## Editorial Note

The “perfect storm” weather conditions of Hurricane Sandy resulted in extensive damage to infrastructure and large flood zones (4). The direct and indirect impacts of the storm led to challenging, and sometimes deadly, conditions for residents, including prolonged power outages, storm surges, and disrupted services. More than half (51.3%) of deaths from Sandy occurred within the first 2 days of the storm, and the most common cause of death was drowning. Approximately half of the drowning deaths were in flooded homes located in areas that were under mandatory evacuation orders as of October 28, 2012, the day before Sandy’s landfall (1).

Before the 1970s, drowning from wind-driven storm surges was by far the most common cause of hurricane-related death (5). Advances in hurricane warning and evacuation systems have helped to reduce drowning deaths. Since that time, hurricanes have had other leading causes of death, such as trauma for the Florida hurricanes in 2004 and 2005, and carbon monoxide poisoning for Hurricane Ike in 2008 (6,7). However, drowning continues to be an important cause of death, and was the leading cause for Hurricane Katrina (2005) and Sandy (8).

The findings in this report are subject to at least two limitations. First, the number of deaths reported is limited to those captured through Red Cross mortality tracking, which is only activated in areas with a Red Cross Disaster Relief Operation. In an evaluation of Red Cross mortality tracking versus Texas’ active disaster-related mortality surveillance during Hurricane Ike, Red Cross had a sensitivity of 47% (Red Cross cases compared with Texas cases) and positive predictive value of 92% (Red Cross Ike cases compared with all Red Cross cases); thus, the cases presented in this report are likely to be actual cases but are unlikely to include all Sandy-related deaths (2). Media sources have reported 131 fatalities in the United States from the storm (9); Sandy mortality statistics, including death certificates, are pending official release. Second, the specific location of death was only available for decedents who died at home, limiting other geographic comparisons. Additionally, New York City’s Evacuation Zones provided the only geographic data available for identifying areas of evacuation; however, 95% of all drowning deaths at home were in or near these areas.

Hurricane-related drowning deaths in evacuation zones are preventable. A successful evacuation depends on officials providing timely messaging to all affected persons, on persons receiving those messages, and on persons having the capacity, resources, and willingness to evacuate. The penetration of evacuation messages to decedents or their communities was not assessed in this report, but future research should evaluate the effectiveness of the hurricane evacuation orders. Given the inability and unwillingness of some residents to evacuate, additional research is needed to identify barriers and motivators for persons during an evacuation and the effectiveness of interventions designed to assist these persons.

## Figures and Tables

**FIGURE 1:**
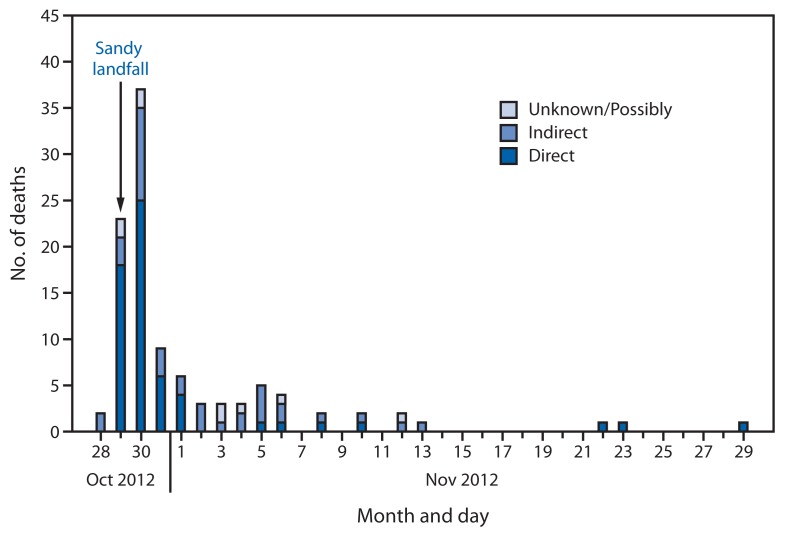
Number of reported deaths related to Hurricane Sandy (direct, indirect, and unknown/possibly), by date — Connecticut, Maryland, New Jersey, New York, Pennsylvania, and West Virginia, October 28–November 30, 2012^*^ ^*^ Excludes deaths with an unknown date of death (n = 12).

**FIGURE 2:**
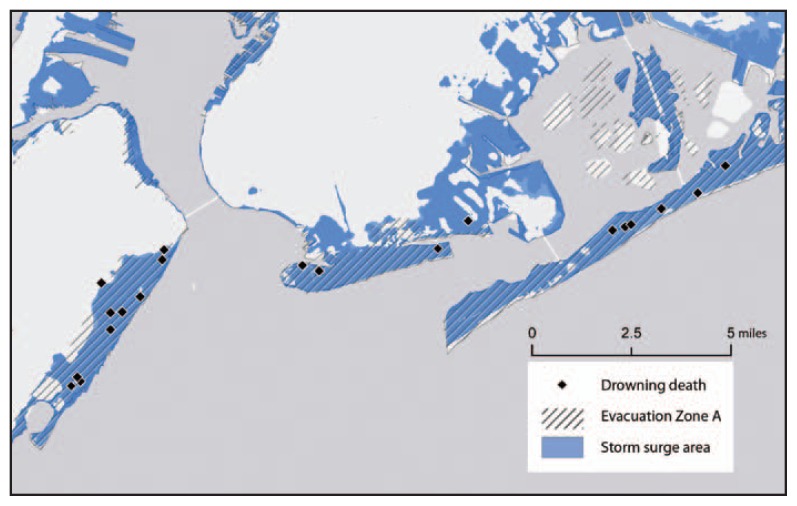
Drowning deaths attributed to Hurricane Sandy that occurred in the decedent’s home (n = 20), in New York state, in relation to the Federal Emergency Management Agency storm surge area and New York City’s Evacuation Zone A — October 28–November 30, 2012

**TABLE:** Characteristics of reported deaths related to Hurricane Sandy for all deaths and drowning deaths — Connecticut, Maryland, New Jersey, New York, Pennsylvania, and West Virginia, October 28–November 30, 2012

	All deaths (N = 117)		Drowning deaths (n = 40)	
		
Characteristic	No.	(%)	No.	(%)
**Age**				
Mean (yrs)	60		59	
Median (yrs)	65		62	
Range (yrs)	1–94		2–90	
Unknown	5	(4.3)	3	(7.5)
**Sex**				
Male	71	(60.7)	26	(65.0)
Female	40	(34.2)	12	(30.0)
Unknown	6	(5.1)	2	(5.0)
**Race**				
White	63	(53.8)	22	(55.0)
Black	15	(12.8)	6	(15.0)
Asian	1	(0.9)	1	(2.5)
Other	8	(6.8)	1	(2.5)
Unknown	30	(25.6)	10	(25.0)
**State (location of death)** *				
New York	53	(45.3)	32	(80.0)
New Jersey	34	(29.1)	4	(10.0)
Pennsylvania	12	(10.3)	0	—
West Virginia	6	(5.1)	0	—
Connecticut	4	(3.4)	1	(2.5)
Maryland	1	(0.9)	0	—
Unknown	7	(6.0)	3	(7.5)
**Source**				
Medical examiner/Coroner	94	(80.3)	38	(95.0)
Family of decedent	10	(8.5)	1	(2.5)
Fire department/Police	4	(3.4)	0	—
Other	3	(2.6)	0	—
Unknown	6	(5.1)	1	(2.5)
**Mechanism of death**				
**Directly related**	67	(57.3)		
Drowning	40	(34.2)		
Trauma-crush/cut/struck	19	(16.2)		
Fall	4	(3.4)		
Motor vehicle	2	(1.7)		
Unknown	2	(1.7)		
**Indirectly related**	38	(32.5)		
Poisoning	10	(8.5)		
Fall	7	(6.0)		
Burn/Electric current	6	(5.1)		
Trauma-crush/cut/struck	5	(4.3)		
Motor vehicle	4	(3.4)		
Other	4	(3.4)		
Unknown	2	(1.7)		
**Unknown/Possibly related**	12	(10.3)		

* p<0.05 between all deaths and drowning deaths.

## Footnotes

* Sandy evolved from a Category 3 hurricane in the Caribbean to an intense post-tropical cyclone before landfall in the United States.
